# Experimental Study on Seismic Performance of Dovetail Profiled Steel Concrete Composite Shear Walls with Self-Tapping Screw Connections

**DOI:** 10.3390/ma18010049

**Published:** 2024-12-26

**Authors:** Zhenfeng Huang, Youwen Tan, Zheng Zhong, Sumei Zhang, Lanhui Guo, Yunhe Wang

**Affiliations:** 1 School of Civil and Environmental Engineering, Harbin Institute of Technology, Shenzhen 518055, China; huangzhenfeng@stu.hit.edu.cn (Z.H.); tanyouwen@stu.hit.edu.cn (Y.T.); 2 Guangdong Provincial Key Laboratory of Intelligent and Resilient Structures for Civil Engineering, Harbin Institute of Technology, Shenzhen 518055, China; 3 School of Science, Harbin Institute of Technology, Shenzhen 518055, China; zhongzheng@hit.edu.cn; 4 School of Civil Engineering, Harbin Institute of Technology, Harbin 150090, China; guolanhui@hit.edu.cn; 5 School of Civil Engineering, Taiyuan University of Technology, Taiyuan 030024, China; wangyunhe@tyut.edu.cn

**Keywords:** composite shear wall, dovetail profiled steel sheet, seismic performance, self-tapping screw connection, design method

## Abstract

To achieve the assembled connection between dovetail profiled steel sheets and the boundary members in dovetail profiled steel concrete composite shear walls (DPSCWs), self-tapping screws were employed. Three DPSCW specimens connected with self-tapping screws were tested under combined axial and cyclic lateral loads to evaluate their hysteretic response, focusing on the influence of the number of self-tapping screws and the axial compression ratio. The self-tapping screw-connected DPSCWs exhibited a mixed failure mode, characterized by shear failure of the profiled steel sheets and compression-bending failure of multiple wall limbs divided by ribs on the web concrete. Except for slight deformation at the screw holes located on the profiled sheets at the corners of the wall, the connections exhibited minimal visible damage. The yield drift ratio of the DPSCW specimens in the test ranged from 1/286 to 1/225, and the ultimate drift ratio ranged from 1/63 to 1/94, both meeting the relevant deformation standards specified in the “Code for Seismic Design of Buildings. Increasing the number of self-tapping screws enhanced the development of local tensile fields on the profiled steel sheets, thereby improving the wall’s load-carrying, deformation, and energy dissipation capacities. However, increasing the axial compression ratio improved the initial stiffness of DPSCWs but reduced their load bearing and deformation capacity. Moreover, a design method for the self-tapping screw connections in DPSCWs was proposed.

## 1. Introduction

**Figure 1:**
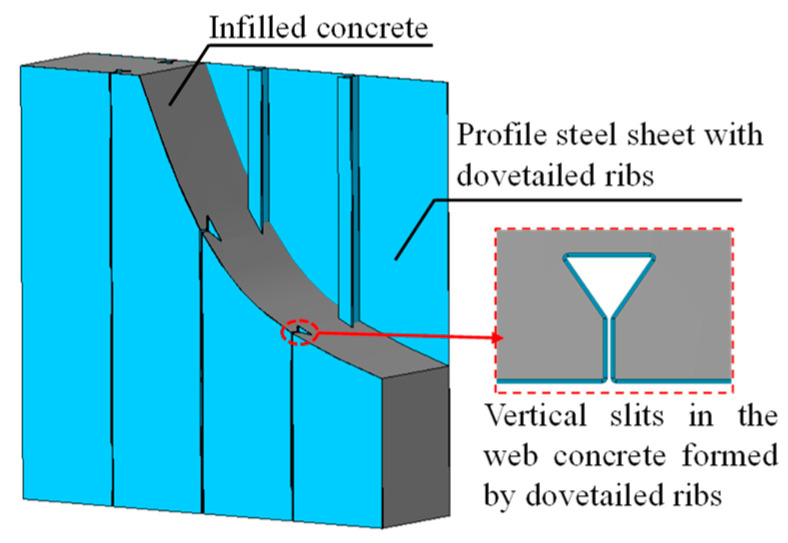
Dovetailed profiled steel concrete composite wall.

Dovetail profiled steel concrete composite shear walls (DPSCWs) are an innovative form of composite shear wall, comprising double layers of dovetail profiled steel sheets filled with concrete (Figure 1). The unique form of the ribs of the profiled steel sheets enables the sheets to work together with concrete without additional shear connectors, avoiding the welding or hole-opening associated with the installation of additional shear connectors in traditional double plate concrete shear walls. Meanwhile, the vertical concealed slits formed by the dovetailed ribs in the web concrete can prevent the penetration of diagonal cracks in the web concrete, thus avoiding the occurrence of brittle shear damage in the web concrete and improving the deformation capacity of the shear wall. Additionally, the dovetail profiled steel sheet has greater out-of-plane stiffness compared with the flat steel plate, which can improve the out-of-plane stability of the dovetail profiled steel sheet under the action of vertical force and reciprocating horizontal load, significantly improve the flexural bearing capacity of the dovetail profiled steel sheet, but also ensures that, during the construction of the shear wall, only a small amount of support can be set up to meet the requirements, which reduces the difficulty of construction. Additionally, profiled steel sheets exhibit significantly higher out-of-plane stiffness than flat steel sheets. This increased stiffness improves their stable load-bearing capacity under vertical and cyclic lateral forces. Furthermore, it enables shear wall construction to achieve out-of-plane stability with minimal support, simplifying the construction process. Therefore, DPSCWs with superior mechanical properties, simple structure, and efficient construction have good application prospects as a lateral force-resisting member in low- and mid-rise buildings.

Uy and Wright et al. [1,2] conducted early experimental studies on the axial compression and compression-bending performance of DPSCWs and performed a theoretical analysis of the compressive buckling behavior of the profiled steel sheets. Based on this, a compression bending capacity calculation formula was proposed considering the post-buckling strength of profiled steel sheet. Tong et al. [3,4] further investigated the overall axial compressive stability, out-of-plane bending, and in-plane eccentric compression performance of DPSCWs using the finite element method based on the experimental data in the above studies, which supplemented and improved the corresponding design methods for closed dovetail profiled steel sheets concrete combined shear walls. Accordingly, the corresponding design method of DPSCWs was supplemented and optimized. Xu et al. [5] conducted experimental research on the seismic performance of DPSCWs with dovetailed ribs arranged in a staggered pattern. The effects of parameters such as axial compression ratio, concrete strength, steel sheet thickness and reinforced connection structure on the seismic performance of DPSCWs were studied. Consequently, the shear bearing capacity calculation formula for the shear wall was developed. Huang and Zhang et al. [6,7,8,9] conducted experimental research on the axial compression and hysteretic performance of DPSCWs. The results showed that the composite action between profiled steel sheets and web concrete can be achieved under the anchorage action provided by the dovetailed rib embedded in the concrete. Combined with finite element analysis, the axial compression and seismic failure mechanism of DPSCWs were revealed, along with the effects of parameters such as thickness ratio, shear span ratio, and axial compression ratio on lateral performance. A simplified calculation method and design recommendations for the axial compression and lateral bearing capacity of DPSCWs were provided.

**Figure 2:**
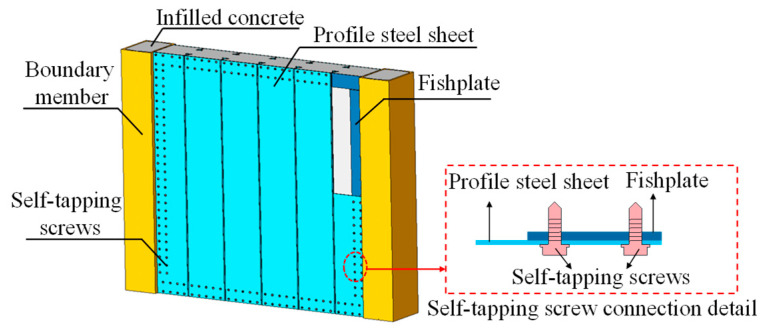
Self-tapping screw-connected DPSCWs.

In the above-mentioned study, the dovetail profiled steel sheets in DPSCWs were welded to the boundary members, and the results confirmed that this welding method provided a reliable connection. However, because the dovetail profiled steel sheets are thin-walled steel sheets with a thickness range of 0.7–2.0 mm, welding operations often cause obvious residual welding deformation near the welded area. Additionally, on-site welding is unsuitable for the requirements of prefabricated construction. Self-tapping screws, known for their high stiffness, bearing capacity, and deformation capability, offer advantages such as flexible installation, ease of operation, and low precision requirements, making them a preferred connection method for thin-walled steel plates [10,11,12,13,14,15,16,17,18,19,20,21]. This paper proposes using self-tapping screws to connect dovetail profiled steel sheets to the boundary members (Figure 2), providing an assembled and effective connection approach for DPSCW. This method addresses the residual deformation issues caused by welding in thin-walled structures and enhances construction flexibility.

Currently, many scholars have conducted studies on the mechanical properties of self-tapping screw connections [10,11,12,13,14,15,16] and thin-walled steel components connected by self-tapping screws [17,18,19,20,21], analyzing the force mechanisms involved and proposing design methods for these connections. However, no research has been conducted on the mechanical performance of DPSCWs connected by self-tapping screws, and their failure mechanism has not yet been clarified, especially the failure characteristics of self-tapping screw connections between thin sheets and thick substrates. Consequently, three DPSCW specimens connected by self-tapping screws were designed and fabricated for quasi-static tests. The influence of the number of self-tapping screws and the axial compression ratio on the failure mode, bearing capacity, strength and stiffness degradation, energy consumption, and deformation capacity of DPSCWs connected by self-tapping screws was analyzed to provide a reference for the seismic design of self-tapping screw-connected DPSCWs.

## 2. Test Program

### 2.1. Specimen Design

**Figure 3:**
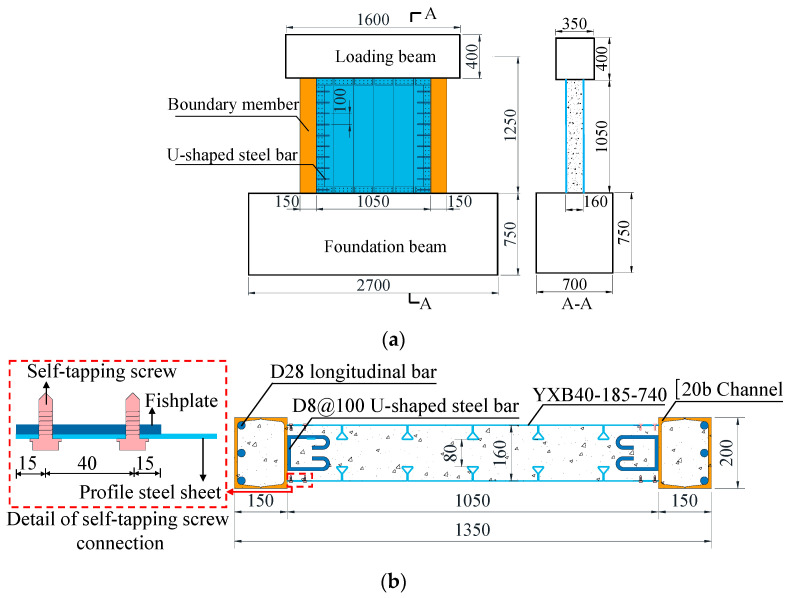
Configurations of specimen. (**a**) Overall Dimensions Schematic. (**b**) Cross-section. (unit: mm).

To ensure that the profiled steel sheets of the shear wall experienced shear failure, which would be the most unfavorable condition for testing the reliability of the self-tapping screw connections, three low shear-span ratio DPSCW with self-tapping screw-connected specimens, labelled DPSCW-S-1~DPSCW-S-3, were designed and tested to examine the cyclic behavior of DPSCW under combined axial and cyclic lateral loads. The parameters studied were the number of self-tapping screws and the axial compression ratio of the wall (Table 1). Figure 3 illustrates the specimen geometry and sectional details. All specimens had the same overall geometry, and each specimen had a barbell-shaped cross-section with an overall width of 1350 mm, which included a 1050 mm wide wall panel composed of double DPSs and concrete infill, as well as two 150 mm × 200 mm rectangular boundary concrete-filled steel tubes (CFSTs). It should be noted that the specimen dimensions were determined with consideration of the capacity of the loading equipment. The wall thickness was set at 160 mm, with 8 mm diameter U-shaped bars welded to the boundary steel tubes to facilitate shear transfer along the interface between the boundary CFSTs and the web concrete, ensuring coordinated deformation and preventing premature failure of the connections. In addition, three HRB400 longitudinal rebars (28 mm in diameter) were placed on each side near the outer web of the boundary CFSTs to enhance the bending capacity of the wall and to ensure that the boundary members did not fail before the wall web.

**Figure 4:**
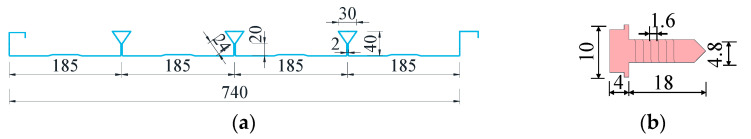
Details of YXB40-185-740 profiled steel sheet and self-tapping screw. (**a**) YXB40-185-740 profiled steel sheet. (**b**) Self-tapping screw. (unit: mm).

**Figure 5:**
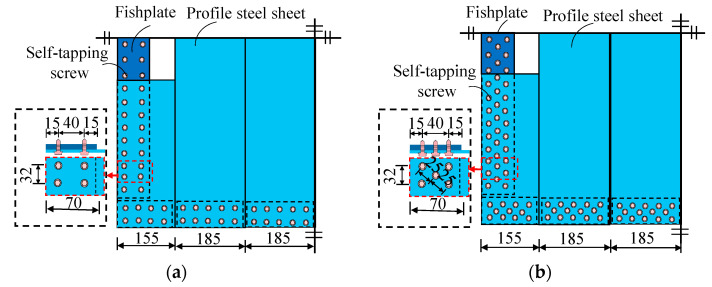
Layout of self-tapping screws. (**a**) Two rows of self-tapping screws. (**b**) Three rows of self-tapping screws. (unit: mm).

In the specimens, commercially available YXB40-185-740 (Xinshijie Coloured Steel Products Co., Wuxi, China) profiled steel sheets with a plate thickness of 1.2 mm (Figure 4) were selected and connected to the 3-mm-thick fishtail plates welded to the boundary members using ST4.8 self-drilling self-tapping screws (Figure 5). It is worth noting that these screws were carefully selected through precise design to ensure they would not fail before the connected steel plates. The technical specifications provided by the manufacturer for the ST4.8 screws are as follows: a diameter of 4.8 mm, a tensile strength of ≥550 MPa, a yield strength of ≥400 MPa, and a hardness range of HRC 20 to 28. The ST4.8 screws are suitable for connecting steel plates with a thickness of up to 3 mm and are available in various lengths to meet specific application requirements. The number of self-tapping screws (56 on one side) for specimen DPSCW-S-1 was determined based on the shear capacity of the profiled steel sheet according to in-plane shear yielding, as shown in Equation (1). These screws were arranged in two rows in accordance with the relevant placement requirements (Figure 5a). Considering the self-tapping screws are also subjected to a certain amount of tensile force after the profiled steel sheets buckling, the number of self-tapping screws of specimen DPSCW-S-1 is increased by approximately 1.4 times and are arranged in three rows according to the plum-shaped arrangement, thus obtaining the arrangement scheme (78 on one side) of specimens DPSCW-S-2 and DPSCW-S-3 (Figure 5b).

(1) $$n=t_{\mathrm{sp}}l_{d}f_{\mathrm{vp}}/N_{v}^{f}$$

where, *t*_sp_ denotes the thickness of the profiled steel sheet; *f*_vp_ denotes the shear strength of the profiled steel sheet; *l*_d_ indicates the width of wall web; 0.8 is the reduction factor applied to self-tapping screws when connecting the ends of the profiled steel sheets to supporting members; and $N_{v}^{f}$ presents the shear strength for individual self-tapping screws, which can be calculated according to Equation (2) with reference to the “Technical Specification for Cold-Formed Thin-Walled Steel Structures” [22].

(2) $$N_{v}^{f}=0.8\times 2.4t_{\mathrm{sp}}df_{\mathrm{up}}$$

where *d* denotes the self-tapping screw diameter and *f*_up_ denotes the tensile strength of the profiled steel sheet.

The designed axial compression ratio of 0.2 and 0.5 was adopted in this study. The relationship between the applied compression load in test and designed axial compression ratio can be seen in Equation (3):

(3) $$n_{d}=\frac{0.8\times 2.4t_{\mathrm{sp}}df_{\mathrm{up}}}{f_{c}A_{c}/1.4+(f_{\mathrm{yp}}A_{p,\mathrm{eff}}+f_{\mathrm{yt}}A_{\mathrm{st}}+f_{\mathrm{yr}}A_{\mathrm{sr}})/1.11}$$

where *n*_d_ represents the design axial load ratio, *f*_c_ denotes the compressive strength of infilled concrete, and *f*_yp_, *f*_yt_, and *f*_yr_ indicate the yield strengths of the profiled steel sheet, boundary steel tube, and longitudinal rebars placed in the boundary members, respectively. *A*_c_, *A*_st_, and *A*_sr_ correspond to the cross-sectional areas of the concrete, boundary steel tubes, and longitudinal rebars, respectively, and *A*_sp,eff_ refers to the effective cross-sectional area of the profiled steel sheets after considering the post-buckling strength, which can be determined using the formula provided in Ref. [7]. Finally, 1.4 and 1.11 are the partial safety factors for concrete and steel materials, respectively.

A reinforced concrete foundation beam with a cross-section of 750 mm × 600 mm was cast integrally with the wall, securing the specimen to the reaction floor. A reinforced concrete loading beam, measuring 400 mm × 350 mm, was also cast to apply vertical and lateral loads to the wall. The effective height (*H*) of the lateral force application point from the top of the foundation beam (Figure 3a) was 1250 mm. Consequently, the shear-span ratio (λ) of these specimens was 0.93, defined as the ratio of the specimen’s effective height to the section width [23,24].

### 2.2. Specimen Fabrication

**Figure 6:**
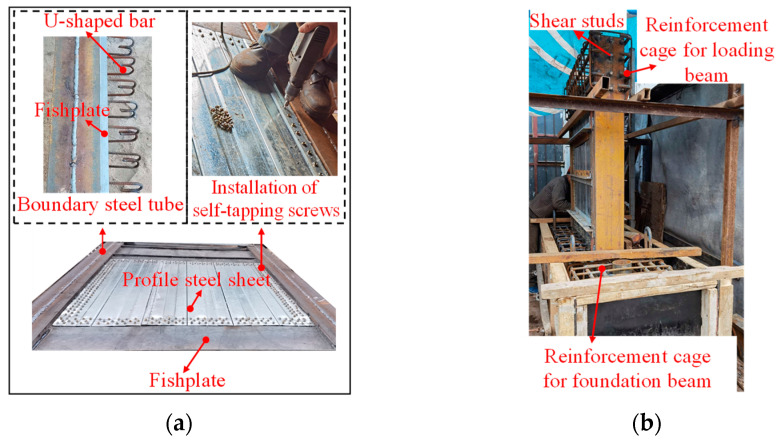

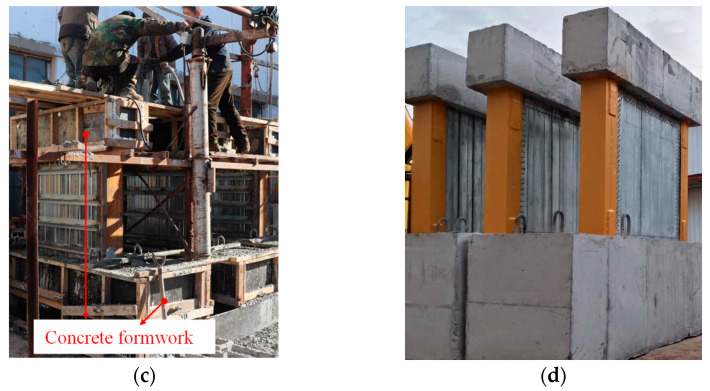
Layout of self-tapping screws. (**a**) Fabrication of steel components. (**b**) Specimen assembly. (**c**) Formwork support and concrete pouring. (**d**) Completed specimens.

The fabrication steps for the specimens are as follows: (1) Weld U-shaped steel bars and fishtail plates onto the side of the boundary steel tube that connects to the profiled steel sheets. Position and assemble welded U-bars and fishtail plate boundary steel tubes with profiled steel plates. Then, secure the profiled steel sheets to the fishtail plates welded to the boundary steel tubes using self-tapping screws, thereby completing the steel component of the wall. (Figure 6a). (2) Tie the reinforcement cages for the loading and foundation beams, and weld sufficient studs onto the sections of the steel wall components embedded within the loading and foundation beams to prevent pull-out. Subsequently, position the steel components within the reinforcement cages and secure them in place (Figure 6b). (3) Pour commercial concrete in the wall after formwork support of the load beams and foundation beams (Figure 6c). The concrete for all specimens was poured in layers during a single session, with a vibrating rod used during the process to ensure proper compaction and density. After pouring, the specimens were cured outdoors under natural conditions. The completed specimens are shown in Figure 6d.

### 2.3. Material Properties

The specimens were made of commercial concrete with a strength grade of C30. During concrete casting, three cubic specimens (150 mm side length) and three prismatic specimens (150 mm × 150 mm × 300 mm) were reserved for each specimen. In accordance with the Chinese standard GB/T 50081-2019 [25], these concrete specimens were tested simultaneously with the main specimen using a 200-ton press. The concrete cubic strength (*f*_cu_), prism strength (*f*_cp_), concrete modulus of elasticity (*E*_c_), and Poisson’s ratio (*ν*_c_) of each specimen were measured and are listed in Table 2. It should be noted that the measured strength of concrete is lower than the nominal strength due to factors such as low winter temperatures during concrete curing.

Three standard tensile coupons were prepared for the profiled steel sheet, fishplate, channel steel, and rebar used in the DPSCW specimens, and the main mechanical property indexes of steel were measured in accordance with the provisions of “Tensile Test of Metallic Materials Part 1: Room Temperature Test Methods” (GB/T228.1-2010) [26]. The average measured results of thickness or diameter, yield strength (*f*_y_), yield strain (*ε*_y_), ultimate strength (*f*_u_), Young’s modulus (*E*_s_), and Poisson’s ratio (*ν*_s_) of these coupons are presented in Table 3.

### 2.4. Test Set-Up and Instrumentation

**Figure 7:**
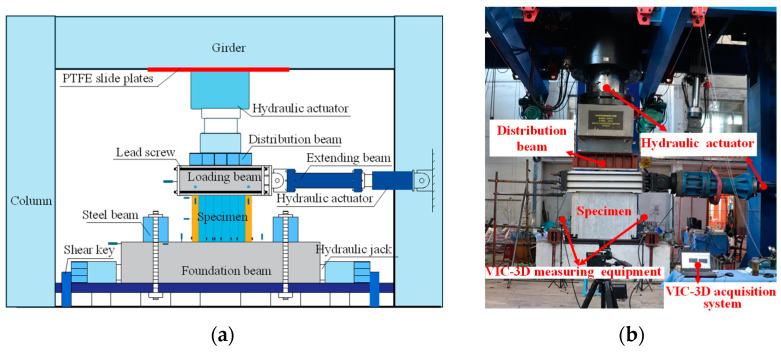
Test setup. (**a**) Schematic of the loading device. (**b**) Photo of loading device.

The test loading was performed on a multifunctional loading device at the Structural Engineering Laboratory of Harbin Institute of Technology (Figure 7). The device had a vertical loading capacity of 30,000 kN and a lateral loading capacity of 3000 kN. The specimen’s foundation beam was anchored to the test platform with ground anchor bolts, and two hydraulic jacks were placed at both ends to prevent sliding. The specimen loading beam was connected to the 300 t horizontal actuator via end plates and lead screws. To ensure uniform distribution of the vertical load across the wall section, a rigid steel beam was positioned between the specimen’s loading beam and the vertical actuators. The vertical actuator, connected to the reaction frame with polytetrafluoroethylene (PTFE) slide plates, was free to move horizontally to accommodate the specimen’s lateral displacement.

**Figure 8:**
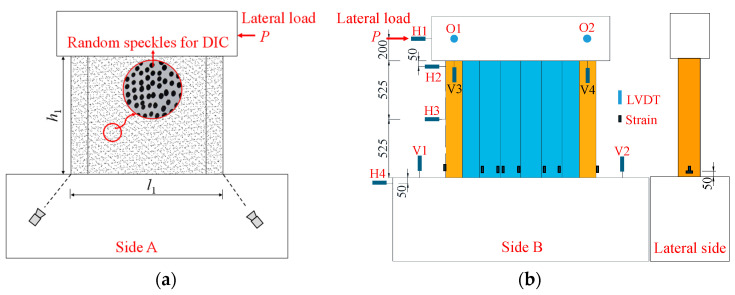
Measurement scheme. (**a**) VIC-3D measurement system. (**b**) Displacement and strain gauges arrangement. (unit: mm).

The measurement system was designed to capture the structural responses of the specimens during the whole loading process (Figure 8). The VIC-3D 8 non-contact full-field strain measurement system, produced by Correlated Solutions, Inc. (CSI), Columbia, SC, USA, was employed for full-field observations of strains and displacements, allowing for the analysis of buckling development in the profiled steel plate. As shown in Figure 8a, the entire front steel surface of the specimens was painted white to enhance the contrast, with random black dots speckled on the steel surface. Linear variable differential transformers (LVDTs) were used to measure displacements at key locations. As displayed in Figure 8b, four LVDTs labeled as H1~H4 were positioned at different heights of the specimens to measure their lateral displacements. Additionally, four LVDTs, labeled as V1~V4, were placed on the loading beam and foundation beam to detect the rotation of the specimens. Strain gauges were installed at important positions to measure the strain development of the steel plate.

### 2.5. Loading Scheme

The loading process followed the Chinese specification JGJ/T 101-2015 [27]. Preloading was first carried out to verify the proper functioning of the loading systems. Compression loads were then applied, beginning at 50% of the expected forces and gradually increasing to 100% in accordance with the axial compression ratio. The compression load was maintained at a constant level during the subsequent lateral cyclic loading phase. The initial loading cycle was force-controlled, with a lateral load of 200 kN. Once the drift ratio reached approximately 1/400, the control mode switched to displacement control. The yield deformation of the specimen was taken as the step length, and each subsequent step consisted of three cycles until the actuator force decreased to 85% of the peak load.

## 3. Test Results and Discussion

### 3.1. Experimental Phenomenon

The specimens exhibited similar failure characteristics under both push and pull loading directions. For clarity, the failure phenomena described here are based on experimental observations during positive loading, when the actuator was pushed to the left (Figure 7).

**Figure 9:**
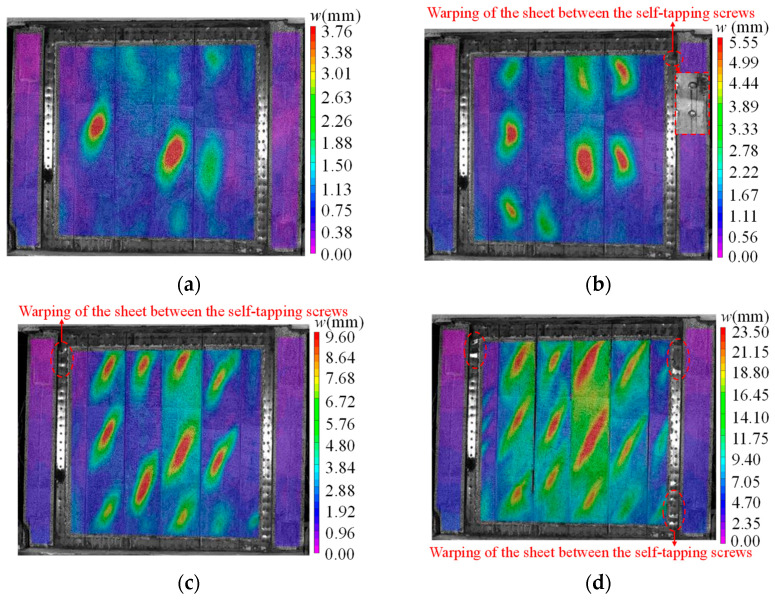
Experimental observations of specimen DPSCW-S-1. (**a**) Initial buckling of DPS. (**b**) Obvious residual deformation. (**c**) Peak load. (**d**) Ultimate displacement.

For the specimen DPSCW-S-1 with two rows of self-tapping screws, no significant changes were observed during the axial compression phase or the initial stage of lateral loading (lateral drift ratio *θ* less than 1/500). When the lateral load *P* reached approximately 1000 kN (0.58 *P*_m_, where *P*_m_ is the peak lateral load), the VIC-3D system captured slight shear buckling on the strip of the steel sheets (Figure 9a), and the sound of bond-slip between the concrete and profiled steel sheets was heard. At this point, the angle between the buckling wave and the loading direction currently is about 45°~50°; after unloading, the buckling wave deformation mostly recovered. As the load increased, new buckling waves continuously formed, the original buckling wavelength extended, and the height of the waves grew. At a lateral load of 1474 kN (0.86 *P*_m_, *θ* = 1/254), 2–3 buckling waves appeared on most strips, except for the third strip from the left. Additionally, slight warping of the steel plate was observed between the two self-tapping screws at the upper right corner of the wall. When the load was reduced to zero, visible residual deformations were observed on the plate strips (Figure 9b), indicating that the specimen had entered a distinct elastic-plastic stage. At 1709 kN (θ = 1/170), the specimen reached the peak lateral load, and the previously formed buckling wave extended into a tensile band, with the angle between the tensile band and the loading direction increasing to 60–65°. Meanwhile, slight warping was observed on the profiled steel sheet between the two self-tapping screws in the upper left corner (Figure 9c). After the peak load, the cyclic lateral load caused further damage and plastic deformation, leading to a gradual reduction in the specimen’s bearing capacity. The tension fields extended and the gaps between the dovetailed ribs widened as the drift ratio increased, but the inclination angle remained stable. Additionally, warping of the profiled steel sheets between the screws at the lower right corner was noted (Figure 9d). During the whole test, the specimen experienced a progression of failure from local shear buckling of the profiled steel sheet strips, followed by the development of tension fields bounded by the ribs embedded in the concrete, no disengagement or shearing of the self-tapping screws was observed, and no loosening was detected when the screws were twisted by hand.

The failure processes of specimen DPSCW-S-2 and specimen DPSCW-S-3 were similar to that of specimen DPSCW-S-1 and will not be repeated here. Notably, the shear buckling of the profiled steel sheets for specimen DPSCW-S-3 was captured by VIC-3D at the load level of 800 kN, indicating that higher axial compression ratio causes the profiled steel sheets of DPSCW to experience shear buckling earlier. In addition, tearing was observed at the turning point of the local tensile field in specimen DPSCW-S-2 and DPSCW-S-3 during the load decrease period.

### 3.2. Failure Mode

**Figure 10:**
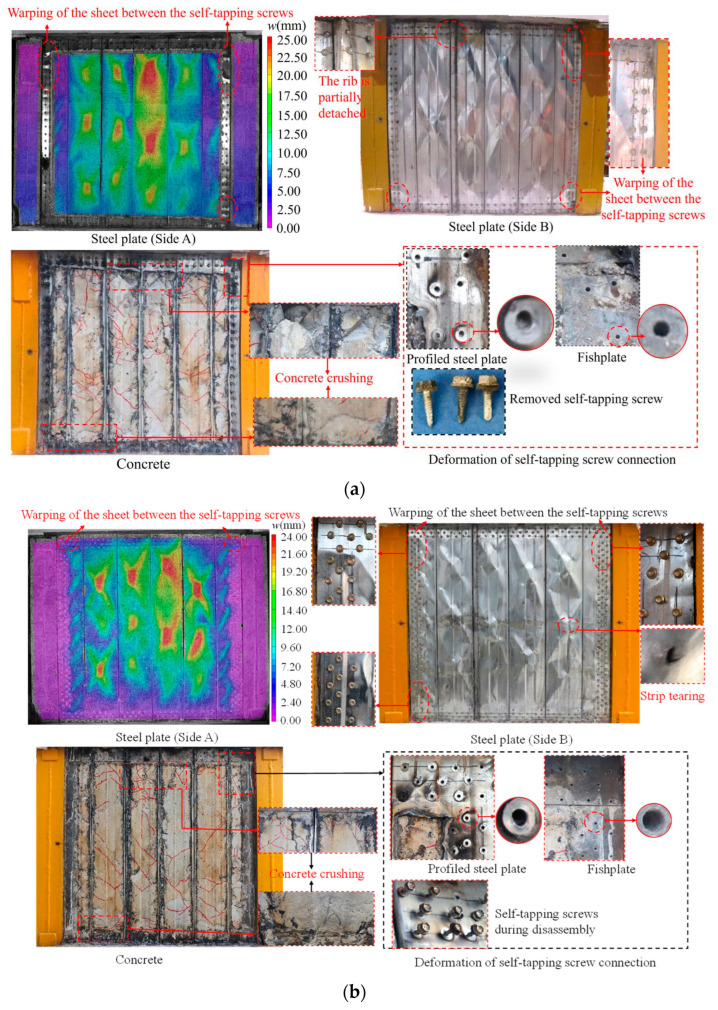

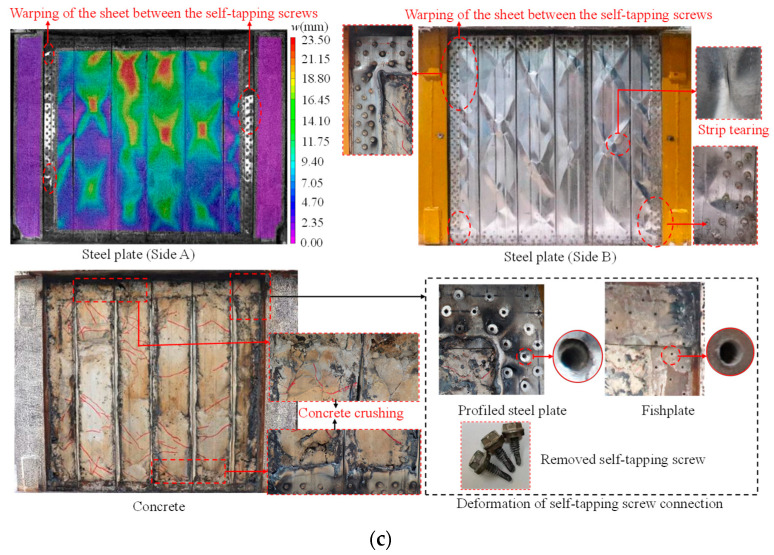
Specimen failure mode. (**a**) Specimen DPSCW-S-1. (**b**) Specimen DPSCW-S-2. (**c**) Specimen DPSCW-S-3.

The overall failure modes of the three specimens are shown in Figure 10. Obvious residual localized tension fields deformation was observed on profiled steel sheets for each specimen, exhibiting significant shear damage. Warping of the steel sheet between the two self-tapping screws was observed around the whole steel sheet, especially at the corners. The warping of the specimen DPSCW-S-1 with two rows of self-tapping screws was more serious than that of the specimens DPSCW-S-2 and DPSCW-S-3 with three rows of self-tapping screws. Additionally, tearing occurred at the turning points of the local tension fields in specimen DPSCW-S-2 and DPSCW-S-3. Apart from the localized warping of the profiled steel sheets between the self-tapping screws, the failure modes of the self-tapping screw-connected profiled steel plates were generally consistent with the failure modes of welded profiled steel plates reported in the existing literature [9]. After removing the steel sheets, there were small, inclined cracks in the concrete column between the ribs, but these inclined cracks were not continuous and did not form a diagonal crack, and concrete crushing was observed at the upper and lower ends of each concrete column between the two ribs. Upon removing the self-tapping screws, it was discovered that the nail holes in the profiled steel near the areas of warping were slightly enlarged, while the holes in the fishtail plate showed no significant deformation. The removed self-tapping screws remained intact.

### 3.3. Lateral Load-Displacement Hysteretic Response

**Figure 11:**
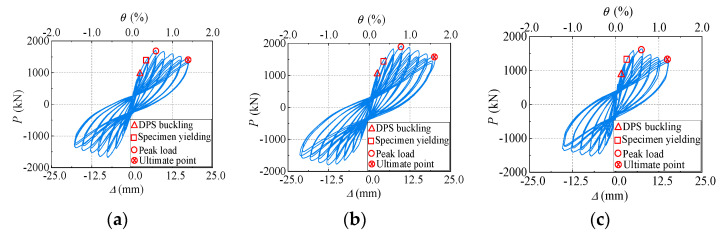
Hysteresis curves. (**a**) Specimen DPSCW-S-1. (**b**) Specimen DPSCW-S-2. (**c**) Specimen DPSCW-S-3.

The lateral load-displacement hysteresis curves of the specimens generally exhibit a reverse “S” shape (Figure 11), with some pinching observed, and the positive and negative loading curves were basically symmetrical. The characteristic points of the first buckling of the DPS, the overall yield of the specimen, the peak load of the specimen, and the yield of the boundary CFSTs for each specimen were pointed at their skeleton curves, where the definition of specimen yielding point is determined from equivalent elastoplastic energy method [28,29]. Before the shear buckling of profiled steel sheets were generated and observed, the specimens showed the linear deformation characteristics, for which the lateral load-displacement curves of loading and unloading were coincident basically with few energy dissipations. With the generation and development of the shear buckling of profiled steel sheets, accompanied by the increases of lateral load, the stiffness of the three specimens decreased obviously, the residual deformation increases, and the area of the hysteresis loop gradually increases. As the horizontal load continued to increase, numerous shear buckling waves were generated and developed on the strips, indicating that the profiled steel sheets exhibit the profound shear deformation under combined axial compression and lateral cyclic loads. Besides, due to the development and closure of concrete cracks under lateral reciprocating loads and the flattening of the buckling wave during unloading and reverse reloading, the hysteresis curve gradually exhibits pinching. After reaching the peak load points, all specimens exhibited a gradual decline in lateral bearing capacity as lateral displacement increased, indicating good deformation capacity.

**Figure 12:**
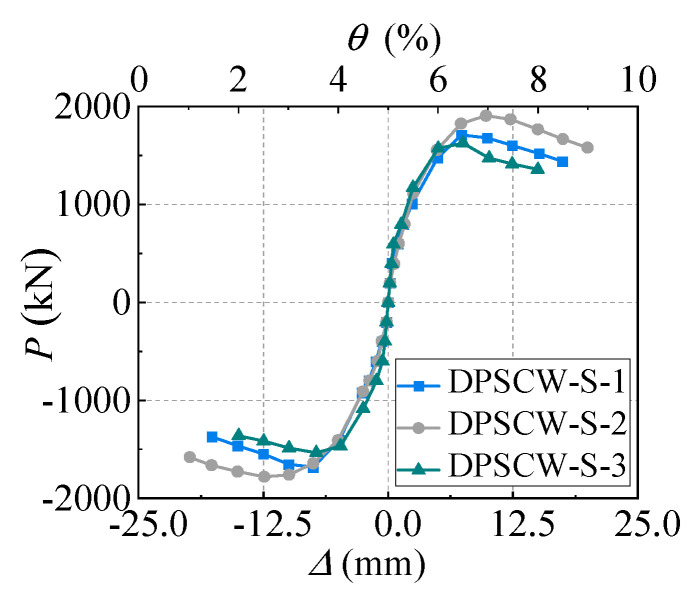
Skeleton curves.

By connecting the peak points of the first cycle at each increment, the skeleton curves for the hysteresis curves were established (Figure 12). The skeleton curves of each specimen are basically symmetric under different loading directions, with the load differences under push and pull loading being at the same displacement level within 5%. Comparative analysis of the envelope curves of specimens DPSCW-S-1 and DPSCW-S-2 showed that the lateral load capacity of DPSCW-S-2 with more self-tapping screws increased from 1695 kN to 1843 kN, which is an increase of 9%, compared to DPSCW-S-1 with fewer screws. However, their initial stiffnesses were similar at 1044 kN/mm and 1066 kN/mm, respectively. For specimens DPSCW-2 and DPSCW-3, which had the same number of self-tapping screws, the axial compression ratios were 0.2 and 0.5, respectively. Increasing the axial compression ratio from 0.2 to 0.5 raised the initial stiffness of the shear wall from 1066 kN/mm to 1289 kN/mm, a 21% increase. However, the load capacity decreased from 1843 kN to 1581 kN, and the load capacity decreased more quickly after the peak load.

### 3.4. Ductility

The ductility of specimens can be evaluated by the displacement ductility ratio (*μ*), which can be calculated through $\mu =\Delta_{u}/\Delta_{y}$; Δ_y_ represents the lateral displacement of yielding and Δ_u_ represents the lateral displacement at which the lateral resistance of specimens decreases by 85% of the peak load. Table 4 lists the horizontal load, drift ratios, and displacement ductility coefficients corresponding to the three characteristic points of yield point, peak point, and limit point of each specimen. The deformation capacity of the self-tapping screw-connected specimen is slightly worse than that of the welded specimen. The average yield drift ratios for specimens DPSCW-S-1, DPSCW-S-2, and DPSCW-S-3 are 1/257, 1/225, and 1/286, respectively, and the average ultimate drift ratios are 1/78, 1/63, and 1/94, respectively. These values exceed the requirements specified in the “Code for Seismic Design of Buildings” (GB 50010-2010) [30], which states that the elastic inter-story drift angle for shear walls should not exceed 1/1000, and the inelastic inter-story drift angle should not exceed 1/120. The ductility coefficients for specimens DPSCW-S-1, DPSCW-S-2, and DPSCW-S-3 are 3.31, 3.51, and 3.09, respectively, all greater than 2, meeting the criteria for moderate ductility failure as defined in the literature [31].

Increasing the number of self-tapping screws by 40% led to a 14% increase in elastic drift ratio and a 23% increase in inelastic drift ratio, with the ductility coefficient rising from 3.31 to 3.51, suggesting improved deformation capacity and ductility with more screws. However, increasing the axial compression ratio from 0.2 to 0.5 decreased the elastic drift ratio by 22%, the inelastic drift ratio by 33%, and the ductility coefficient from 3.51 to 3.09, reflecting diminished deformation capacity. Although the load-bearing capacity of the unconnected specimens did not significantly decrease, its deformation capacity failed to meet the relevant code requirements.

### 3.5. Stiffness and Strength Degradation

**Figure 13:**
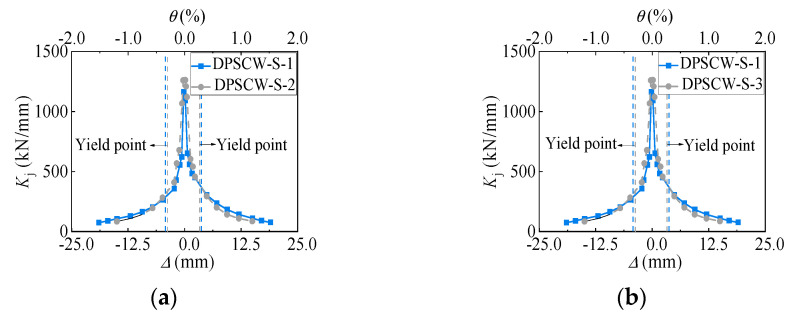
Stiffness degradation curves. (**a**) Specimen DPSCW-S-1. (**b**) Specimen DPSCW-S-2.

The development of cracks as well as the accumulation of inelastic deformations due to the development of local tensile fields in the profiled steel sheet under cyclic loading can result in the gradual deterioration of stiffness and strength, which can assess the damage degree of the DPSCWs connected by self-tapping screws. Referring to Chinese code JGJ/T 101-2015 [27], the secant stiffness *K*_j_, which is defined in Equation (4), is used to depict stiffness degradation (Figure 13). At the initial loading stage, all specimens experienced significant stiffness reduction, which followed an exponential distribution as lateral displacement increased. After yielding, the stiffness degradation of each specimen gradually slowed and stabilized. Under the same axial compression ratio, the stiffness degradation curves of specimen DPSCW-S-2, with more self-tapping screws, nearly coincided with those of specimen DPSCW-S-1, which had fewer screws, up to the peak load. Beyond the peak load, DPSCW-S-2 exhibited a slower rate of stiffness degradation compared to DPSCW-S-1. With a higher axial compression ratio, the initial stiffness of specimen DPSCW-S-3 increased from 1066 kN/mm to 1289 kN/mm, a 21% improvement. However, the cyclic stiffness degradation rate was faster and more pronounced in specimens with a higher axial compression ratio.

(4) $$K_{j}=\frac{\left|+P_{j}\right|+\left|-P_{j}\right|}{\left|+\Delta_{j}\right|+\left|-\Delta_{j}\right|}$$

where +*P*_j_ and −*P*_j_ represent the peak lateral loads at j_th_ loading level under push and pull loading, respectively; +Δ_j_ and −Δ_j_ represent the corresponding lateral displacement associated with +*P_j_* and −*P_j_*, respectively.

**Figure 14:**
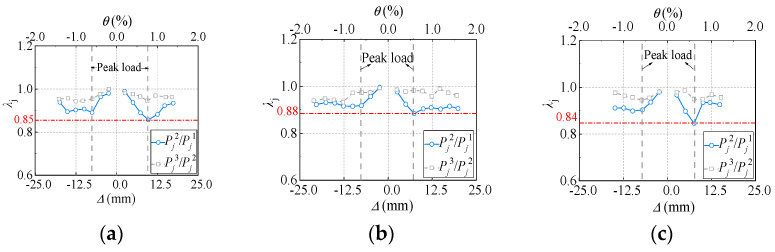
Strength degradation curve. (**a**) Specimen DPSCW-S-1. (**b**) Specimen DPSCW-S-2. (**c**) Specimen DPSCW-S-3.

Referring to Chinese code JGJ/T 101-2015 [27], determination of the strength degradation ratio $\lambda_{j}$ can be calculated using Equation (5), where a lower coefficient value indicates greater strength loss. As shown in Figure 14, before peak load, the strength degradation index of all specimens gradually decreased from 0.97–1.0 to approximately 0.84–0.88. After the peak load, the strength degradation index exhibits fluctuations but all larger than 0.9. This indicates that the load-bearing capacity of self-tapping screw-connected DPSCWs is generally stable, and that the self-tapping screw connections can ensure cooperative performance between the profiled steel sheets and boundary members. Additionally, it was observed that the strength degradation index during the third cycle of repeated loading was higher than that during the second cycle, indicating that the damage caused by the third loading cycle was less severe than that of the second at the same drift angle.

(5) $$\lambda_{j}=\frac{{P_{j}}^{i+1}}{{P_{j}}^{i}}$$

where *P*_j_^i^ represents the peak lateral load of *i*_th_ load cycle under the *j*_th_ load level and *P*_j_^i+1^ is the peak lateral load of (*i +* 1)_th_ load cycle under the *j*_th_ load level.

Overall, the DPSCW specimens connected by self-tapping screws show relatively stable strength and stiffness degradation behavior, indicating that the self-tapping screw connection ensures effective cooperation between the profiled steel plate and the boundary members under seismic loads. The reliability of the self-tapping screw connection has been validated.

### 3.6. Energy Dissipation

**Figure 15:**
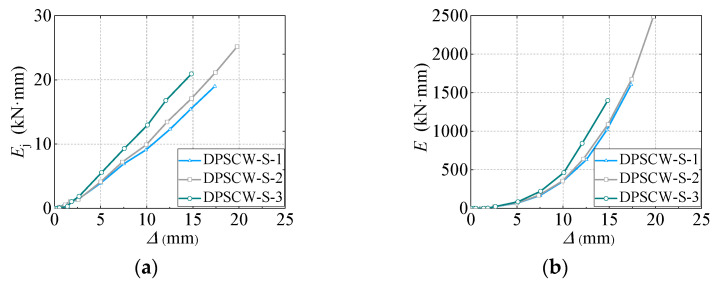
Energy dissipation curve. (**a**) Dissipated energy per load cycle. (**b**) Cumulative dissipated energy.

The enclosed area (${E_{j}}^{i}$) of hysteresis curves can be used to evaluate the energy dissipation of DPSCWs under cyclic loadings. Specifically, at the same deformation level, the larger the area enclosed by the average single-cycle hysteresis curve (${E_{j}}^{i}=\sum_{i=1}^{n}{E_{j}}^{i}/n$), the greater the energy dissipated per cycle, indicating higher energy dissipation efficiency. The greater the total area of all hysteresis loops ($\sum E_{j}=\sum_{j=1}^{m}\sum_{i=1}^{n}{E_{j}}^{i}$) of the specimen before failure, the larger the total energy dissipated, reflecting the specimen’s stronger energy dissipation capacity. Higher energy dissipation efficiency and capacity are beneficial for improving the seismic performance of self-tapping screw-connected DPSCWs. Figure 15 illustrates the average energy dissipation per cycle (*E*_j_) and the cumulative energy dissipation($\sum E_{j}$) at each displacement level of the test specimens. Initially, both the average energy dissipation per cycle and cumulative energy dissipation of the specimen are small. As the local buckling of the profiled steel plate occurred, the specimens entered the elastoplastic stage, significantly increasing energy dissipation. At the same displacement level, DPSCW-S-2, with three rows of self-tapping screws, exhibited 55% higher cumulative energy dissipation (2486 kN·mm) compared to DPSCW-S-1, which had two rows (1605 kN·mm). This indicates that increasing the number of self-tapping screws around the profiled steel sheet enhances the restraining effect of the screws on the sheet, leading to more fully developed localized tension filed along the sheet strips, and the deformation of the specimen and the ability to dissipate energy were improved. Specimen DPSCW-S-3, which had a higher axial compression ratio, exhibited greater average and cumulative energy dissipation than specimen DPSCW-S-2, which had a lower axial compression ratio, at the same displacement level. However, due to the poorer deformability of specimen DPSCW-S-3 with a larger axial compression ratio than that of specimen DPSCW-S-2, the final cumulative total energy dissipation of DPSCW-S-3 (1399 kN·mm) is 44% less than that of specimen DPSCW-S-2 (2486 kN·mm). This suggests that although increasing the axial compression ratio enhances per-cycle energy dissipation, it diminishes overall deformability, resulting in lower total cumulative energy dissipation. Therefore, the axial force applied to concrete composite shear walls with self-tapping screw-connected dovetail profiled steel sheets should be carefully managed.

## 4. Design Method for the Self-Tapping Screw Connections in DPSCWs

Based on the test analysis results of DPSCWs connected using self-tapping screws, the failure mode of specimen DPSCW-S-1, designed according to the shear capacity of the profiled steel sheet, was similar to that of specimen DPSCW-S-2, in which the number of self-tapping screws was increased by approximately 1.4 times compared to DPSCW-S-1 and arranged in a plum blossom pattern across three rows. However, the deformation of the self-tapping screw holes on the profiled steel sheet of the specimen DPSCW-S-1 is more obvious, and the ribs of the sheet are partially detached. Furthermore, the load-bearing capacity and deformation ability of specimen DPSCW-S-1 were both inferior to those of specimen DPSCW-S-2. This indicates that the number of self-tapping screws calculated using the design method adopted during the specimen design should be insufficient. Therefore, the design method for the lateral resistance and connections of self-tapping screw-connected profiled steel plates in DPSCWs should be developed based on their failure mechanisms.

**Figure 16:**
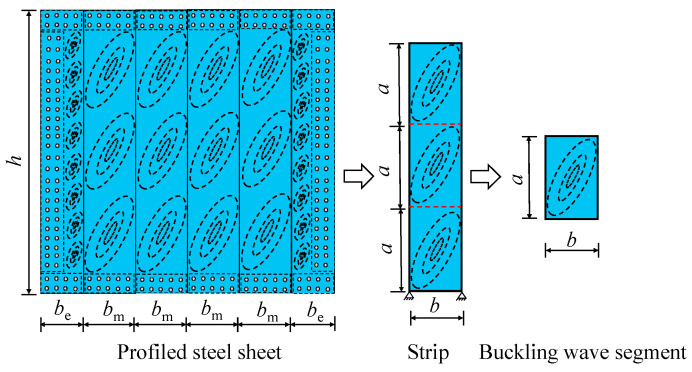
Simplified analytical model for profile steel sheet.

Experimental studies on DPSCWs with self-tapping screw connections showed that failure of the dovetail profiled steel sheets is characterized by local buckling and the development of extended tension fields on the strips. Additionally, within the studied range of axial compression ratios, calculations based on the von Mises yield criterion show that the influence of axial stress on the shear capacity of the profiled steel sheets is less than 5.2%. Therefore, the effect of axial stress on the profiled steel sheets is negligible, and the overall shear capacity of the profiled steel sheets can be approximated as the shear response of multiple independent strips. Due to the periodic occurrence of buckling waves along the length of the profiled steel strips, the shear behavior of each strip can further be approximated as the shear response of a single tension band segment (Figure 16).

**Figure 17:**
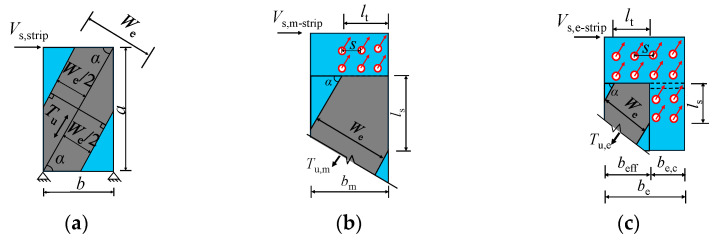
Simplified analytical model for self-tapping screw connections of profile steel sheets. (**a**) Effective strip method model. (**b**) Self-tapping screw force analysis in middle strip. (**c**) Self-tapping screw force analysis in edge strip.

Referring to the North American specification AISI S400-15 [32,33], the effective strip method was employed to calculate the shear capacity of the tension band segment. Figure 17a illustrates the effective strip method model for a single tension band segment. In Figure 17a, *a* represents the height of a single tension band segment on the profiled steel sheet’s strip and *b* denotes the width of the tension band segment; the length *a* is twice the width b, as determined from the local tension bands observed in the test. The angle α, defined as the angle between the lateral and diagonal directions of the tension band segment, is given by α = arctan(*a*/*b*). *T*_u_ represents the load carrying capacity of the effective strips. *W*_e_ denotes the width of the effective strips, which can be calculated according to the following formula [34,35]:

(6) $$W_{e}=\left\{\begin{array}{l} W_{\max},\;\;\;\;\lambda \leq 0.0819\;\\ \rho W_{\max}\;\;\;\lambda >0.0819 \end{array}\right.$$

(7) $$\rho =\frac{1-0.55{\left(\lambda -0.08\right)}^{0.12}}{\lambda^{0.12}}$$

(8) $$\lambda =\frac{1.736\alpha_{1}\alpha_{2}}{\beta_{1}\beta_{2}{\beta_{3}}^{2}\psi }$$

where *W*_max_ represents the maximum effective strip width, calculated as *b*/sinα. *ρ* is the discount factor of effective strip width. λ is the correction factor that accounts for component thickness, material properties, self-tapping screw spacing, aspect ratio, and other factors of the profiled steel sheet. *α*_1_, *α*_2_ are the correction factors considering the difference of materials, *α*_1_ = *f*_up_/310.3, *α*_2_ = *f*_uf_/310.3, with *f*_up_ and *f*_uf_ being the ultimate tensile strength of the material of the profiled steel sheet and the fishtail plate, respectively. *β*_1_, *β*_2_ are the correction coefficients considering the difference in the thickness of the members, defined as *β*_1_ = *t*_sp_/0.457, *β*_2_ = *t*_sf_/0.457, with *t*_sf_ being the thicknesses of the fishtail plate; *β*_3_ is the correction coefficient considering the spacing and rows of the tapping screws, given by *β*_3_ = (2*R* × *s*)/152.4, where *R* and *s* are the rows and self-tapping screw spacing of the self-tapping screws, respectively; ψ represents the height-to-width ratio of individual tension strip segments.

Based on the effective strip model for the single tension band segment, the analytical model of the self-tapping screw force within the effective strip width on the middle strips and edge strips was given in Figure 17b,c, so that the shear bearing capacity of the intermediate and edge strips can be obtained as:

(9) $$V_{s,m-\mathrm{strip}}=T_{u,m}\times \cos\alpha$$

(10) $$V_{s,e-\mathrm{strip}}=T_{u,e}\times \cos\alpha$$

where *T*_u,m_ and *T*_u,e_ are the bearing capacity of the effective strip for the middle and edge strips, respectively, as calculated in Equations (11) and (12):

(11) $$T_{u,m}=\min(P_{n,m},\frac{W_{e}}{2}t_{\mathrm{sp}}f_{\mathrm{yp}})+\frac{W_{e}}{2}t_{\mathrm{sp}}f_{\mathrm{yp}}$$

(12) $$T_{u,e}=\min(P_{n,e},\frac{W_{e}}{2}t_{\mathrm{sp}}f_{\mathrm{yp}})$$

where *P*_n,m_ and *P*_n,e_ represent the shear bearing capacities of the self-tapping screws within the effective strip width of the middle and edge strips, respectively.

Based on the arrangement and stress analysis of self-tapping screws within the effective strip width presented in Figure 17, the formulas for calculating the shear bearing capacities *P*_n,m_ and *P*_n,e_ of these self-tapping screws in the tension band sections on the intermediate and edge strips are as follows:

(13) $$P_{n,m}=(\frac{W_{e}}{2s\sin\alpha }R-\xi )N_{v,b}^{f}$$

(14) $$P_{n,e}=(\frac{W_{e}}{2s\sin\alpha }R-\xi )N_{v,b}^{f}+(\frac{W_{e}}{2s\cos\alpha }R-\xi )N_{v,b}^{f}+n_{c}N_{v,b}^{f}$$

where $N_{v,b}^{f}$ is the shear bearing capacity of a single self-tapping screw, which can be calculated using Equation (2); *ξ* is equal to 0 when the self-tapping screws are arranged side by side, and 1 when arranged in plum blossom shape; *n*_c_ represents the number of self-tapping screws at the corners of the edge strips with the effective strips.

Adopting Equations (9)–(14), the formula for calculating the shear capacity of self-tapping screw-connected profiled steel sheets in DPSCWs can be obtained:

(15) $$V_{s}=2V_{s,e-\mathrm{strip}}+mV_{s,m-\mathrm{strip}}$$

where *m* is the number of middle strips.

**Figure 18:**
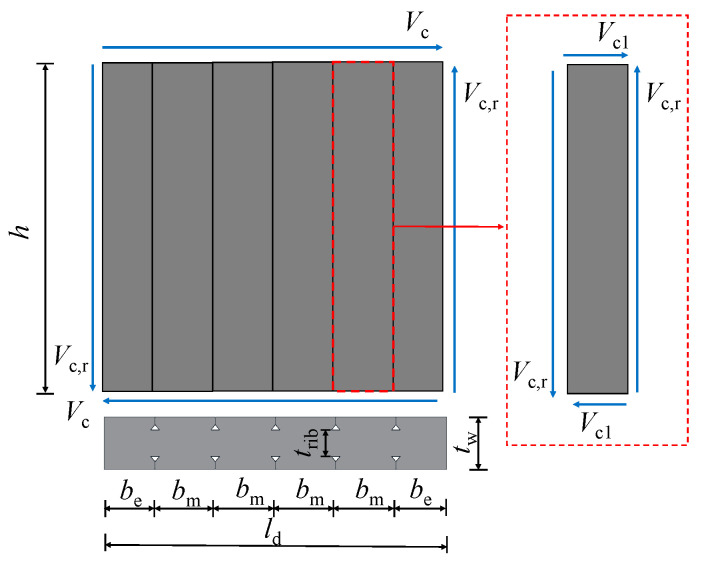
Analytical model for web concrete.

The shear capacity of the profiled steel sheets for the test specimen can be determined by subtracting the loads carried by the web concrete and boundary members from the total wall capacity obtained from the test. The lateral resistance of the web concrete (*V*_cn_) is calculated using the simplified model shown in Figure 18, as described in reference [8], and is given by Equations (16)–(18).

(16) $$V_{c}=2\frac{V_{c,r}b_{e}}{h}+m\frac{V_{c,r}b_{m}}{h}$$

(17) $$V_{r,c}=0.15f_{c}t_{\mathrm{rib}}h$$

(18) $$V_{\mathrm{cn}}=0.15f_{c}t_{\mathrm{rib}}l_{d}+0.13N$$

where *V*_r,c_ is the shear strength of the connecting concrete; *h* is the height of the wall; *b*_e_ and *b*_m_ are the widths of the middle and edge concrete wall limbs divided by dovetailed ribs, respectively; and *t*_rib_ is the thickness of the rib-area-concrete. *N* is the compressive axial load applied to the wall, which should be less than 0.2 *f*_c_*A*_w_, and *A*_w_ is the cross-sectional area of the wall.

For the boundary members in shear walls, which primarily resist the overturning moment of the wall [8], their lateral resistance is mainly provided by the boundary member on the compressive side, as specified in Code for Design of Composite Structures (JGJ 138-2016) [36]:

(19) $$V_{b}=0.5f_{t}l_{b}t_{\mathrm{bw}}+0.4f_{yst}A_{st}$$

where *f*_t_ and *f*_yst_ is the tensile strength of the concrete and the yield strength of the boundary tube, respectively, *l*_b_ and *t*_bw_ are the width and thickness of the boundary member, respectively, and *A*_st_ signifies the cross-sectional area of a single boundary steel tube.

In summary, the shear capacity of the profiled steel sheets for the test specimen can be calculated using the following formula:

(20) $$V_{s,\exp}=P-0.15f_{c}t_{\mathrm{rib}}l_{d}-0.13N-4f_{v,b}l_{b}t_{\mathrm{sb}}$$

The shear capacity *V*_s,cal_ of the profiled steel sheets was calculated for each specimen using Equation (19) and is presented in Table 5, along with the test shear capacity values *V*_s,exp_ obtained from Equation (16). The error between the calculated and test results is within 12%, demonstrating that the proposed calculation method effectively predicts the shear capacity of profiled steel sheets in the DPSCWs connected by self-tapping screws.

Based on the above analysis, to ensure a reliable connection of the profiled steel sheets, the number of self-tapping screws within the effective strip width of the middle and edge strips must satisfy the following requirements:

(21) $$n_{m-\mathrm{strip}}\geq \frac{W_{e,\max}t_{\mathrm{sp}}f_{\mathrm{yp}}}{2N_{v,b}^{f}}$$

(22) $$n_{e-\mathrm{strip}}\geq \frac{W_{e,\max}t_{\mathrm{sp}}f_{\mathrm{yp}}}{N_{v,b}^{f}}$$

Based on the calculated number of self-tapping screws needed within the effective strip width, the screws should be arranged within the effective width range (as shown in the *l*_t_ and *l*_s_ ranges in Figure 16) according to the relevant requirements. For design purposes, the arrangement of self-tapping screws outside the effective width range should be consistent with the arrangement within the effective width range.

## 5. Conclusions

This study experimentally investigated the seismic behavior of self-tapping screw-connected DPSCWs, focusing on the effects of varying the number of self-tapping screws and axial compression ratios. The following conclusions were drawn:

(1) The self-tapping screw-connected DPSCW specimens exhibited a failure progression starting with local shear buckling of the strips, followed by inclined tension fields anchored by the concrete-embedded ribs. The wall concrete experienced compression-bending failure in several concrete columns, while the self-tapping screw connections remained intact with no significant damage.
(2) The specimens achieved yield drift ratios of 1/286 to 1/225, ultimate drift ratios of 1/63 to 1/94, and ductility coefficients of 3.09 to 3.51, all meeting the deformation capacity requirements in the “Code for Seismic Design of Buildings”. This confirms the effectiveness of self-tapping screw connections in ensuring reliable force transfer between the profiled steel plates and boundary members.
(3) Increasing the number of self-tapping screws by 40% slightly increased peak load capacity by 9.5%, ultimate drift ratio by 23%, and ductility factor by 6%, with minimal impact on initial stiffness.
(4) Increasing the axial compression ratio from 0.2 to 0.5 resulted in a 13% increase in initial stiffness. However, the peak load capacity, ultimate drift ratio, and ductility coefficient decreased by 14%, 33%, and 12%, respectively.
(5) An analytical model for the shear capacity of profiled steel sheets connected by self-tapping screws in DPSCWs was developed based on the effective strip method, with the error between the calculated and experimental results within 12%.

In future studies, a comparative analysis will be conducted on the seismic performance of self-tapping screw-connected DPSCWs and welded-connected DPSCWs to evaluate the adaptability and applicability of different connection methods. Additionally, finite element analyses will be performed on the mechanical behavior of self-tapping screw-connected DPSCWs, accompanied by systematic parametric studies. Based on the results of the finite element analyses, the proposed design methodology will be refined to enhance its accuracy and reliability.

## Figures and Tables

**Table 1:** Details of specimens.

Specimen	Number of Self-Tapping Screws	Design Axial Compression Ratio	Applied Axial Force *N* (kN)
DPSCW-S-1	56 (2 rows)	0.20	2000
DPSCW-S-1	78 (3 rows)	0.20	2000
DPSCW-S-3	78 (3 rows)	0.50	5000

**Table 2:** Concrete properties.

Specimen	*f*_cu_ (MPa)	*f*_cp_ (MPa)	*E*_c_ (GPa)	*ʋ*_c_
DSCW-S-1	28.25	26.33	27.9	0.19
DSCW-S-2	30.07	28.87	29.8	0.20
DSCW-S-3	27.11	22.63	26.9	0.20

**Table 3:** Mechanical properties of steel elements.

Components	Measured Thickness or Diameter (mm)	*f*_y_ (MPa)	ε_y_	*f*_u_ (MPa)	*E*_s_ (GPa)	*ʋ*_s_
YXB40-185-740	1.17	331	0.0018	382	200	0.28
Fishplate	2.96	325	0.0017	372	195	0.29
Channel steel	8.95	341	0.0018	490	199	0.26
Longitudinal rebar	27.40	467	0.0023	648	182	-

**Table 4:** Comparison of experimental results.

Specimen	Load Direction	Initial Stiffness	Yield of DPSCW			Peak Lateral Load			Ultimate Displacement			Ductility Coefficient
		*K*_0_/(kN/mm)	*P*_y/_kN	Δ_y/_mm	*θ*_y_	*P*_m/_kN	Δ_m_/mm	*θ*_m_	*P*_u/_kN	Δ_u/_mm	*θ*_u_	*μ* = Δ_u_/Δ_y_
DPSCW-S-1	Puch	1063	1454	4.80	1/263	1709	7.30	1/172	1453	16.18	1/78	3.38
	Pull	1025	1438	4.94	1/250	1680	7.52	1/167	1428	15.97	1/78	3.23
	Average	1044	1446	4.87	1/257	1695	7.41	1/170	1441	16.07	1/78	3.31
DPSCW-S-2	Puch	1072	1617	5.48	1/227	1876	9.94	1/125	1628	19.52	1/64	3.56
	Pull	1061	1507	5.89	1/213	1810	12.62	1/99	1587	20.35	1/61	3.45
	Average	1066	1562	5.69	1/225	1843	11.28	1/111	1608	19.94	1/63	3.51
DPSCW-S-3	Puch	1340	1385	4.14	1/294	1627	7.5	1/167	1391	13.05	1/94	3.15
	Pull	1238	1355	4.49	1/278	1535	7.09	1/175	1348	13.58	1/93	3.02
	Average	1289	1370	4.32	1/286	1581	7.30	1/167	1369	13.32	1/94	3.09

**Table 5:** Comparison between the calculated and tested shear capacity of profiled steel sheets.

Specimen	Tested Lateral Load *P*_m_ (kN)	Tested Shear Capacity of Profiled Steel Sheets *V*_s,exp_ (kN)	Calculated Shear Capacity of Profiled Steel Sheets *V*_s,cal_ (kN)	*V*_s,cal_/*V*_s,exp_
DPSCW-S-1	1695	359	331	0.92
DPSCW-S-2	1843	437	389	0.89
DPSCW-S-3	1581	346	389	1.12
			Average	0.98

## Data Availability

The original contributions presented in this study are included in the article. Further inquiries can be directed to the corresponding author.
